# No Boundaries for Toxicology in Clinical Medicine: One Health, One Society and One Planet for All of Us

**DOI:** 10.3390/jcm12082808

**Published:** 2023-04-11

**Authors:** Ricardo Jorge Dinis-Oliveira

**Affiliations:** 1 TOXRUN—Toxicology Research Unit, University Institute of Health Sciences (IUCS), CESPU, 4585-116 Gandra, Portugal; ricardo.dinis@iucs.cespu.pt or ricardinis@med.up.pt; Tel.: +351-224-157-216; 2 Department of Public Health and Forensic Sciences, and Medical Education, Faculty of Medicine, University of Porto, 4200-319 Porto, Portugal; 3 UCIBIO-REQUIMTE, Applied Molecular Biosciences Unit, Laboratory of Toxicology, Department of Biological Sciences, Faculty of Pharmacy, University of Porto, 4050-313 Porto, Portugal; 4 MTG Research and Development Lab, 4200-604 Porto, Portugal

The concept of One Health is not new; it can be traced back for at least two hundred years [1], firstly as One Medicine and One World and then One Health, following the emergence of severe acute respiratory disease (SARS) in early 2003. There is no single, technical agreed definition of One Health. Despite several having been proposed, they typically point to a broad discipline by covering several scientific domains such as environment, ecosystem, domestic and wildlife animals, human health, social sciences, and biodiversity, among others. The most used definition shared by the US Centers for Disease Control and Prevention and the One Health Commission establishes One Health “as a collaborative, multisectoral, and transdisciplinary approach—working at the local, regional, national, and global levels—with the goal of achieving optimal health outcomes recognizing the interconnection between people, animals, plants, and their shared environment”. The One Health Global Network states that “One Health recognizes that the health of humans, animals and ecosystems are interconnected. It involves applying a coordinated, collaborative, multidisciplinary and cross-sectoral approach to address potential or existing risks that originate at the animal–human-ecosystems interface”. Another definition is provided by the One Health Institute of the University of California at Davis, which considers One Health as an “approach to ensure the well-being of people, animals and the environment through collaborative problem solving—locally, nationally, and globally”. For the World Health Organization, One Health “is an approach to designing and implementing programs, policies, legislation and research in which multiple sectors communicate and work together to achieve better public health outcomes”. Irrespective of the definition considered, the One Health concept clearly focuses on consequences, responses, and actions at the animal–human–ecosystem interface (Figure 1) [2,3]. In other words, interdisciplinary collaboration is at the heart of the One Health concept since health problems are frequently complex and require multifactorial mitigation strategies to be effectively solved. What is interesting to note is that while veterinarian professionals have easily adopted the One Health concept, the medical community has been much slower to recognize its spirit, despite efforts of different bodies, such as the World Health Organization. In the field of toxicology and pharmacology, and as previously suggested for the medical community to reach the “physician of the future” [4,5], the One Health concept should be discussed and trained in curricular plans to highlight that public health and the mitigation of toxic insults cannot be achieved without analyzing the triad of One Health. Launched in 2016, the One Health Day, officially commemorated on the 3rd of November, has been progressively adopted by different countries around the world and the awareness of its value in understanding phenomena such as emerging zoonotic infectious diseases, neglected tropical diseases, antimicrobial resistance, climate change, environmental health and pollution, and food safety, among others, has been steadily increasing. Already in March 2023, due to the health emergencies such as COVID-19 pandemic, monkeypox, Ebola outbreaks, and continued threats of other zoonotic diseases, food safety, antimicrobial resistance, as well as ecosystem and climate negative alterations, the heads of the Quadripartite organizations working on One Health (i.e., World Health Organization (WHO), Food and Agriculture Organization of the United Nations (FAO), United Nations Environment Programme (UNEP) and the World Organisation for Animal Health (WOAH)), at their first annual meeting, issued an unprecedented call for an accelerated and prioritized global political action focusing on One Health as a unique solution to face future treats [6]. 

Historically focused on zoonoses [7], the concept of One Health has paid less attention to animal, human, and environmental exposure to toxics. The application of the One Health concept to toxicology and pharmacology is obvious, as these specialists on xenobiotics work in synergy with other medical and nonmedical professionals. Despite the variability in the dose response and toxicokinetics between domestic and wildlife animals, plants, and humans, biochemical and physiological systems are highly conserved among species, meaning that the toxic effects can be common to them [8]. This suggests that, if a specific group of species is at risk, other groups of species can be also affected. Therefore, the term “One Toxicology” emerged to encourage more efficient collaborative research, data, and knowledge sharing, as well as stewardship actions among groups of toxicologists [8]. Some examples of One Health toxicology research include studies on the effects of pollution on aquatic ecosystems, the impact of pesticides on honeybee populations and other pollinators, and the health risks associated with exposure to toxics trafficked via foods. In addition, some tragedies highlight the concept of One Health applied to toxicology. The Minamata Bay (Japan) tragedy in the 1950s, due to mercury exposure, is one example since it illustrated how an industrial pollutant can be biomagnified in an aquatic ecosystem from marine microorganisms up the food chain, and how the poisoned and dead floating fish, predators, and scavengers should have served as sentinels and alarm for this outbreak of environmental illness, before its effects were felt by the human population [9]. The great London fog, which blanketed the British capital for five days in December 1952, caused more than 12,000 human deaths [10]. The etiology was only discovered almost one year later, but respiratory-related sudden death in cattle was indicative of the air pollution problem before it was recognized as the cause of human deaths [11]. Nevertheless, animals can only act as sentinels for human beings (as occurred in the case of canaries used to warn coal miners of methane or carbon monoxide levels) if their existence is maintained. Another good example was described in Rachel Carson’s book *Silent Spring*, reporting the tragedy of silenced birds due to biomagnification and exposure to the organochlorine insecticide dichlorodiphenyltrichloroethane (DDT) [12]. What is unquestionable is that thousands of chemicals are produced and disseminated through manufacturing, pharmacological treatments, pest control, and food industries, to name few examples. This means that there is an interdependence between humans and the ecosystems, highlighting that human health is ultimately dependent on the health of our planet [13,14]. The European Union developed the REACH Regulation (focused on four processes: the registration, evaluation, authorization, and restriction of chemicals) to address the safety of chemicals already in the marketplace, aiming to protect human health and the environment. The REACH Regulation places responsibility on the industry to manage the risks from chemicals and to provide safety information on the substances. It also requests the progressive substitution of the most dangerous chemicals (referred to as “substances of very high concern”) when suitable alternatives have been identified. In the US, the Toxics Substances Control Act (TSCA) Reform offers similar approaches.

In the toxicology–pharmacology areas, some recent findings published in the Journal of Clinical Medicine, with an intersection with other scientific areas, are worth mentioning. The everyday appearance of new psychoactive substances (NPS) and the consequent morbidity and mortality associated with their consumption is a highly recognized problem [15]. Piperazine derivatives are among the most studied psychoactive compounds in this group of designer drugs, especially as alternatives to the recreational use of 3,4-methylenedioxymethamphetamine and other amphetamine derivatives. However, for the success of effective treatments, analytical techniques are needed, since laboratories do not have or include toxicological analysis in their portfolio, rendering diagnosis mostly based on clinical history. Welz et al., in complementary studies [16,17], developed two liquid chromatographic techniques using a diode-array detector (LC-DAD) and a mass spectrometer (LC-MS) for the rapid qualitative and quantitative analysis of piperazine derivatives in serum and urine. Such techniques represent an important evolution to facilitate the clinical diagnosis of the intoxications by these substances. Additionally, on the subject of NPS, Poyatos and colleagues [18], following the PRISMA (preferred reporting items for systematic reviews and meta-analyses) guidelines, presented for the first time a systematic review on the origins, chemistry, pharmacodynamics, and pharmacokinetics of cathinones such as mephedrone, methylone, and diethylpropion. It was evidenced that these psychoactives induce a range of desirable and reinforcing effects that may, to some extent, result in abuse potential. However, psychoactives, even illicit ones, can also possess useful biological activities if their dose is correctly titrated, as I recently highlighted in an Editorial of a new journal of the Multidisciplinary Digital Publishing Institute (MDPI) portfolio [19]. Following this desideratum, toxicological risks and clinical benefits of ayahuasca intake were fully reviewed, exploring the direct link between decoction doses and corresponding blood concentrations and their physiological and psychological effects in humans [20]. In this context of the abuse of psychoactives, Daniel Dacosta-Sánchez and colleagues [21] took a step forward by exploring the usefulness of electronic health records (EHRs) and Real World Data to uncover the factors that may explain the dissatisfaction with therapy, increasing the risk of drop-out in patients with opioid and cannabis use disorder (SUD) [22,23]. Particularly, their data complement the evidence previously obtained through observational studies and clinical trials by having access to a large sample size (*n* = 2458 inpatients).

As highlighted before for several pharmacological drugs with abuse potential [24,25,26,27,28,29,30,31,32], the study of drug pharmacokinetics and pharmacodynamics represents a powerful approach to prevent adverse effects such as dependence by understanding the consequences of the polymorphic metabolism and implementing the most suitable doses and posology. In this sense, a crossover, double-blind, placebo-controlled, randomized clinical trial to characterize the pharmacokinetics and pharmacodynamics of carisoprodol and its main active metabolite (i.e., meprobamate) was developed with the aim of uncovering the possible factors that may contribute to carisoprodol’s dependence and abuse [33,34]. Indeed, carisoprodol is a centrally acting muscle relaxant, but it has been used for non-medical reasons, including diversion and trafficking [35]. The most relevant findings were the implications of CYP2C19 genotype’s polymorphism in the metabolism of carisoprodol, and the lack of evidence of withdrawal symptoms and a reduced risk of dependence [33,34]. In a different field, a protocol following the STROBE guideline was proposed to study the pharmacokinetic properties of piperacillin/tazobactam and hydrocortisone, as well as the concentrations of inflammatory markers and adrenal steroids during the treatment of community-acquired pneumonia [36]. It is expected that a personalized therapeutic regimen and improved treatment outcomes will be possible due to high inter- and intra-individual variability in pharmacokinetic parameters.

Discussing toxicology in a unifying approach to balance and optimize the health of people, animals, and the environment is needed. By taking a One Health approach to toxicology, researchers can gain a more complete understanding of the impacts of toxic substances on the health of multiple species, as well as the ecological and societal implications of these impacts, and clues to reach a greener chemistry. This can help inform policy decisions concerning the regulation and management of toxic substances, with the goal of promoting the health and well-being of all living beings. Innovative research directly related to toxicology (e.g., ecotoxicology, education in toxicology, food toxicology, environmental toxicology, analytical toxicology, clinical toxicology, forensic toxicology, veterinary toxicology, occupational toxicology, toxinology and regulatory toxicology), but also at the interface of biomedical, biochemistry and environmental Sciences (e.g., medicine, nutrition, veterinary medicine, pharmaceutical sciences, and forensic sciences), with cross-cutting issues within the Sustainable Development Goals, is welcome. Real world data and evidence will further improve the translational impact, enabling the development of ecological and needs-oriented research, and to identify the specificities, risk, and resilience factors of the individuals to the toxic insults on health. Finally, multidisciplinary approaches involve the study of toxicological interactions and implications to One Health, biosorbents and environment, food microbiology, chemical compounds and therapeutic implications, drug repurposing, biomarkers, cancer studies, the development of analytical methodologies, and the analysis of real-world data and evidence for a better understanding of the diseases from a global picture point of view.

## Figures and Tables

**Figure 1:**
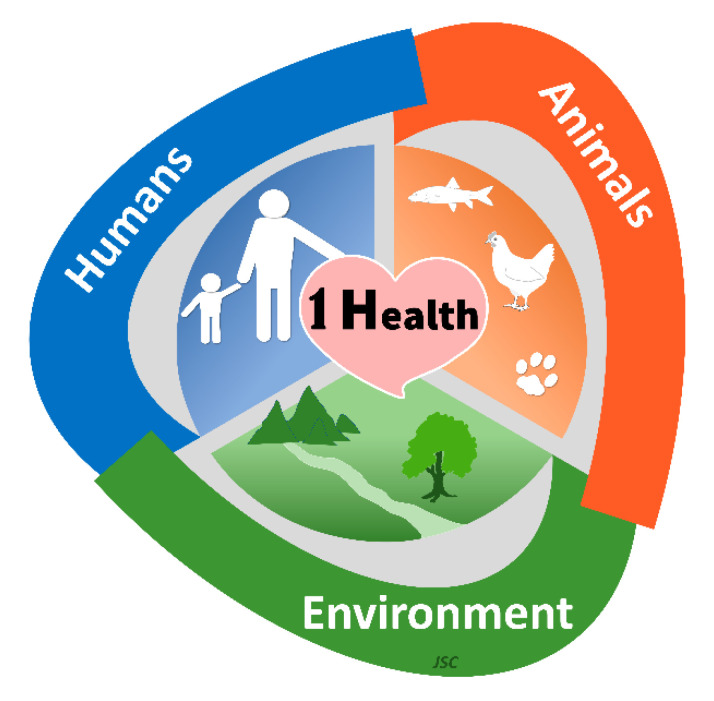
The tridimensional concept of One Health toxicology. Artwork is a gift from Professor João Soares Carrola (JSC) of the University of Trás-os-Montes and Alto Douro.

## References

[1] Atlas R.M. (2013). One Health: Its origins and future. Curr. Top Microbiol. Immunol..

[2] Garcia S.N., Osburn B., Cullor J. (2019). A one health perspective on dairy production and dairy food safety. One Health.

[3] Hoelzer K., Wong N., Thomas J., Talkington K., Jungman E., Coukell A. (2017). Antimicrobial drug use in food-producing animals and associated human health risks: What, and how strong, is the evidence?. BMC Vet. Res..

[4] Rabinowitz P.M., Natterson-Horowitz B.J., Kahn L.H., Kock R., Pappaioanou M. (2017). Incorporating one health into medical education. BMC Med. Educ..

[5] Gómez A., Balsari S., Nusbaum J., Heerboth A., Lemery J. (2013). Perspective: Environment, biodiversity, and the education of the physician of the future. Acad. Med..

[6] World Health Organization Quadripartite Call to Action for One Health for a Safer World. https://www.who.int/news/item/27-03-2023-quadripartite-call-to-action-for-one-health-for-a-safer-world.

[7] Kahn L.H., Kaplan B., Steele J.H. (2007). Confronting zoonoses through closer collaboration between medicine and veterinary medicine (as ‘one medicine’). Vet. Ital..

[8] Beasley V. (2009). ‘One toxicology’, ‘ecosystem health’ and ‘one health’. Vet. Ital..

[9] Rumbeiha W.K. (2012). Toxicology and “One Health”: Opportunities for Multidisciplinary Collaborations. J. Med. Toxicol..

[10] Polivka B.J. (2018). The Great London Smog of 1952. Am. J. Nurs..

[11] Buttke D.E. (2011). Toxicology, environmental health, and the “One Health” concept. J. Med. Toxicol..

[12] Jarman W.M., Ballschmiter K. (2012). From coal to DDT: The history of the development of the pesticide DDT from synthetic dyes till Silent Spring. Endeavour.

[13] King L.J., Anderson L.R., Blackmore C.G., Blackwell M.J., Lautner E.A., Marcus L.C., Meyer T.E., Monath T.P., Nave J.E., Ohle J. (2008). Executive summary of the AVMA One Health Initiative Task Force report. J. Am. Vet. Med. Assoc..

[14] Whitmee S., Haines A., Beyrer C., Boltz F., Capon A.G., de Souza Dias B.F., Ezeh A., Frumkin H., Gong P., Head P. (2015). Safeguarding human health in the Anthropocene epoch: Report of The Rockefeller Foundation-Lancet Commission on planetary health. Lancet.

[15] Wadsworth E., Drummond C., Deluca P. (2018). The Dynamic Environment of Crypto Markets: The Lifespan of New Psychoactive Substances (NPS) and Vendors Selling NPS. Brain Sci..

[16] Welz A., Koba M., Kośliński P., Siódmiak J. (2021). Rapid Targeted Method of Detecting Abused Piperazine Designer Drugs. J. Clin. Med..

[17] Welz A., Koba M., Kośliński P., Siódmiak J. (2022). Comparison of LC-MS and LC-DAD Methods of Detecting Abused Piperazine Designer Drugs. J. Clin. Med..

[18] Poyatos L., Torres A., Papaseit E., Pérez-Mañá C., Hladun O., Núñez-Montero M., de la Rosa G., Torrens M., Fuster D., Muga R. (2022). Abuse Potential of Cathinones in Humans: A Systematic Review. J. Clin. Med..

[19] Dinis-Oliveira R.J. (2022). The Genesis of a New Open-Access Journal Focused on the Latest Scientific Advances in Psychoactive Substances. Psychoactives.

[20] Nižnanský Ľ., Nižnanská Ž., Kuruc R., Szórádová A., Šikuta J., Zummerová A. (2022). Ayahuasca as a Decoction Applied to Human: Analytical Methods, Pharmacology and Potential Toxic Effects. J. Clin. Med..

[21] Dacosta-Sánchez D., Díaz-Batanero C., Fernandez-Calderon F., Lozano Ó.M. (2021). Impact of Cluster B Personality Disorders in Drugs Therapeutic Community Treatment Outcomes: A Study Based on Real World Data. J. Clin. Med..

[22] Marsch L.A., Campbell A., Campbell C., Chen C.H., Ertin E., Ghitza U., Lambert-Harris C., Hassanpour S., Holtyn A.F., Hser Y.I. (2020). The application of digital health to the assessment and treatment of substance use disorders: The past, current, and future role of the National Drug Abuse Treatment Clinical Trials Network. J. Subst. Abuse Treat.

[23] Magalhães T., Dinis-Oliveira R.J., Taveira-Gomes T. (2022). Digital Health and Big Data Analytics: Implications of Real-World Evidence for Clinicians and Policymakers. Int. J. Environ. Res. Public Health.

[24] Costa R., Oliveira N.G., Dinis-Oliveira R.J. (2019). Pharmacokinetic and pharmacodynamic of bupropion: Integrative overview of relevant clinical and forensic aspects. Drug Metab. Rev..

[25] Dinis-Oliveira R.J. (2018). Metabolic Profiles of Propofol and Fospropofol: Clinical and Forensic Interpretative Aspects. Biomed. Res. Int..

[26] Dinis-Oliveira R.J. (2019). Metabolism and metabolomics of opiates: A long way of forensic implications to unravel. J. Forensic. Leg. Med..

[27] Dinis-Oliveira R.J., Pereira C.L., da Silva D.D. (2019). Pharmacokinetic and Pharmacodynamic Aspects of Peyote and Mescaline: Clinical and Forensic Repercussions. Curr. Mol. Pharmacol..

[28] Lobato-Freitas C., Brito-da-Costa A.M., Dinis-Oliveira R.J., Carmo H., Carvalho F., Silva J.P., Dias-da-Silva D. (2021). Overview of Synthetic Cannabinoids ADB-FUBINACA and AMB-FUBINACA: Clinical, Analytical, and Forensic Implications. Pharmaceuticals.

[29] Nóbrega L., Dinis-Oliveira R.J. (2018). The synthetic cathinone α-pyrrolidinovalerophenone (α-PVP): Pharmacokinetic and pharmacodynamic clinical and forensic aspects. Drug Metab. Rev..

[30] Oliveira N.G., Dinis-Oliveira R.J. (2018). Drugs of abuse from a different toxicological perspective: An updated review of cocaine genotoxicity. Arch. Toxicol..

[31] Silva A.R., Dinis-Oliveira R.J. (2020). Pharmacokinetics and pharmacodynamics of dextromethorphan: Clinical and forensic aspects. Drug Metab. Rev..

[32] Sousa A., Dinis-Oliveira R.J. (2020). Pharmacokinetic and pharmacodynamic of the cognitive enhancer modafinil: Relevant clinical and forensic aspects. Subst. Abus..

[33] Calvo A., Alonso S., Prieto E., Ascaso-Del-Rio A., Ortuño J., Fernandez N., Portolés A. (2022). Single and Multiple Dose PK-PD Characterization for Carisoprodol. Part I: Pharmacokinetics, Metabolites, and 2C19 Phenotype Influence. Double-Blind, Placebo-Controlled Clinical Trial in Healthy Volunteers. J. Clin. Med..

[34] Calvo A., González-Hidalgo M., Terleira A., Fernández N., Portolés A. (2022). Carisoprodol Single and Multiple Dose PK-PD. Part II: Pharmacodynamics Evaluation Method for Central Muscle Relaxants. Double-Blind Placebo-Controlled Clinical Trial in Healthy Volunteers. J. Clin. Med..

[35] Li Y., Delcher C., Brown J.D., Wei Y.J., Reisfield G.M., Winterstein A.G. (2019). Impact of Schedule IV controlled substance classification on carisoprodol utilization in the United States: An interrupted time series analysis. Drug Alcohol Depend..

[36] Vincze I., Czermann R., Nagy Z., Kovács M., Neely M., Farkas R., Kocsis I., Karvaly G.B., Kopitkó C. (2022). Assessment of Antibiotic Pharmacokinetics, Molecular Biomarkers and Clinical Status in Critically Ill Adults Diagnosed with Community-Acquired Pneumonia and Receiving Intravenous Piperacillin/Tazobactam and Hydrocortisone over the First Five Days of Intensive Care: An Observational Study (STROBE Compliant). J. Clin. Med..

